# Microbial gardening in the ocean's twilight zone: Detritivorous metazoans benefit from fragmenting, rather than ingesting, sinking detritus

**DOI:** 10.1002/bies.201400100

**Published:** 2014-09-12

**Authors:** Daniel J Mayor, Richard Sanders, Sarah L C Giering, Thomas R Anderson

**Affiliations:** 1) Institute of Biological and Environmental Sciences, Oceanlab, University of AberdeenAberdeen, UK; 2) National Oceanography Centre, University of SouthamptonSouthampton, UK

**Keywords:** carbon cycling, detritus, mesopelagic, microbial loop, nutrition, polyunsaturated fatty acid, zooplankton

## Abstract

Sinking organic particles transfer ∼10 gigatonnes of carbon into the deep ocean each year, keeping the atmospheric CO_2_ concentration significantly lower than would otherwise be the case. The exact size of this effect is strongly influenced by biological activity in the ocean's twilight zone (∼50–1,000 m beneath the surface). Recent work suggests that the resident zooplankton fragment, rather than ingest, the majority of encountered organic particles, thereby stimulating bacterial proliferation and the deep-ocean microbial food web. Here we speculate that this apparently counterintuitive behaviour is an example of ‘microbial gardening’, a strategy that exploits the enzymatic and biosynthetic capabilities of microorganisms to facilitate the ‘gardener's’ access to a suite of otherwise unavailable compounds that are essential for metazoan life. We demonstrate the potential gains that zooplankton stand to make from microbial gardening using a simple steady state model, and we suggest avenues for future research.

## Introduction: Carbon sequestration in the oceans via biological activity

The biological carbon pump (BCP) refers to the suite of processes that store atmospheric CO_2_ in the deep ocean via the production of organic matter in the upper ocean and its sinking and subsequent remineralisation at depth. Between 5 and 12 gigatonnes of photosynthetically-fixed carbon leaves the sunlit (euphotic) waters of the upper ocean each year via sinking particles 1. Typically <10% of this organic matter reaches the deep-seafloor: the vast majority is remineralised to inorganic carbon by the respiration of the organisms that reside in the dimly lit waters of the ‘mesopelagic zone’, also known as the ‘twilight zone’. This begins at the base of the euphotic zone, where photosynthesis is no longer possible, and extends down to ∼1,000 m. The depth at which remineralisation occurs plays a major role in determining the size of biological carbon storage in the oceans, and hence the oceanic role in global climate regulation 2. Understanding the factors influencing the strength of the BCP remains a major goal of biological oceanography.

The twilight zone ecosystem contains a diverse range of prokaryotes and eukaryotes. The microbial web begins with free-living and particle-attached heterotrophic bacteria. These organisms support active communities of heterotrophic flagellates and ciliates, which can be two to four orders of magnitude more abundant on particles than in the surrounding water 3. The metazoan component of the twilight zone can be broadly categorised as (a) temporary residents that migrate into the cool, deep waters during daylight hours to reduce their metabolic demands and minimise predation pressure from visual predators, and (b) true residents. Feeding in surface waters under the cover of darkness provides temporary residents with sufficient resources to support their daily metabolic demands, eliminating the need for them to feed in the mesopelagic 4. By contrast, the resident metazoan community, the biomass of which is dominated by copepods ≤1 mm 4,5, tiny crustaceans that swim with rowing-like movements of their fore-legs, must derive their energetic and nutritional requirements at depth through detritivory and/or carnivory.

The collective respiration of organisms resident in the twilight zone should, at steady state, equal the loss of carbon estimated as the reduction in flux as it sinks down through the mesopelagic. Until recently, attempts to demonstrate this balance have failed (e.g. 6). A balanced budget has, however, now successfully been obtained based on field observations and a modelling study of the twilight zone at the Porcupine Abyssal Plain (PAP) Site (49°N 16° 30′W) in the North Atlantic 4. A central conclusion of this work is the understanding that whilst the resident microbial and metazoan communities each intercept approximately 50% of the sinking organic matter, the metazoans fragment, rather than ingest, most of the fast-sinking particles that they encounter. The resulting production of smaller, slowly- or non-sinking organic particles stimulates bacterial proliferation and the microbial food web. The low growth efficiencies of bacteria 7 and protozoans 8 necessitates that much of the organic carbon that enters this pathway is ultimately respired. Thus, the carbon budget constructed for the PAP site showed that respiration in the twilight zone is dominated by the bacteria (79%), and zooplankton account for only a minor fraction (21%) 4.

**Figure 1:**
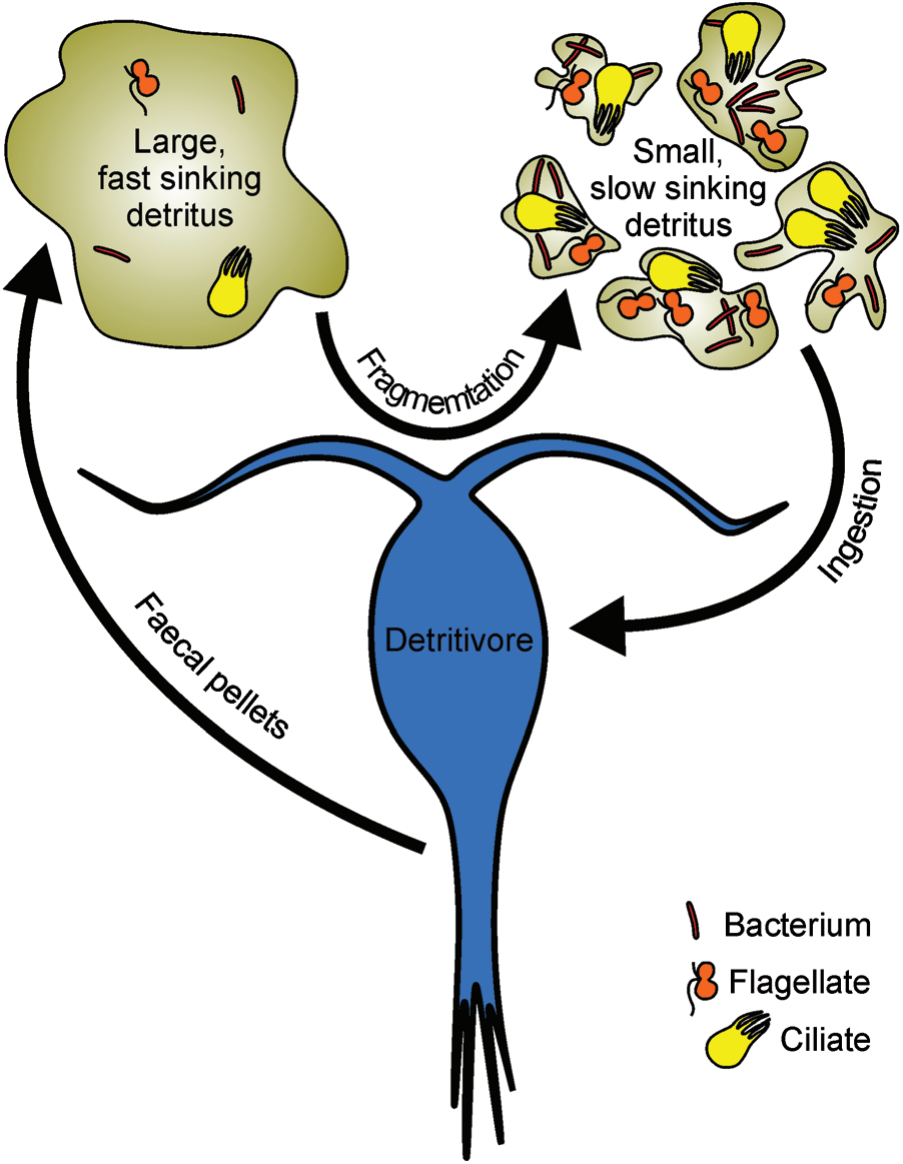
Microbial gardening in the twilight zone. We speculate that detritivorous zooplankton stimulate the production of labile and nutritious microbial biomass by fragmenting, rather than ingesting, large detrital particles. Detritus fragmentation increases the amount of organic matter exposed to bacterial degradation, encouraging their population growth. Increased bacterial biomass fuels the growth of flagellates and ciliates and the concomitant production of essential biochemical compounds. These protists, which are energetically and nutritionally superior to detritus, are sufficiently large to be effectively harvested by the zooplankton. Image not to scale.

The question thus arises, why are detritivorous zooplankton such messy feeders, apparently breaking up and releasing sinking organic matter, rather than ingesting it directly as food for processing in the gut? This could reflect a simple necessity; some detrital particles, e.g. the houses of larvaceans (free-swimming tunicates) and the faecal pellets of euphausiids (krill), are far too large to be directly ingested. Here we develop an alternative explanation for why this seemingly wasteful behaviour occurs. The average sinking detrital particle within the twilight zone, which is considerably reworked by zooplankton 9, is difficult to digest, contains little in the way of nutrition and hence is a poor substrate for metazoan growth. We speculate that deliberate fragmentation of large detrital particles, which stimulates its transformation into smaller particles rich in microbial biomass, is a means by which zooplankton increase the energetic and nutritional content of particulate organic matter for subsequent ingestion (Fig. 1). This is conceptually analogous to the ‘microbial gardening’ observed in coastal benthic detritivores 10,11. An early investigation into microbial gardening 10 demonstrated that apparently detritivorous estuarine amphipods actually consume the detritus-associated microbial community; plant residues pass through the animals undigested. Amphipods encourage, or garden, microbial biomass by fragmenting detritus, causing the numbers of particle-associated bacteria, flagellates, and ciliates to increase rapidly and greatly. Nevertheless, a trade-off exists between ingesting a high-quantity, low-quality detrital diet and a low-quantity, high-quality diet rich in microbes. Here, we articulate the case for microbial gardening as a strategy for maximising fitness in resident mesopelagic zooplankton, and use a simple food web model to demonstrate that it can be an effective growth strategy.

## Life in the twilight zone

Twilight zone residents experience hydrostatic pressures of up to 100 bar and temperatures as low as 4 °C. They also face considerable spatial and temporal variability in the distribution of organic substrates, reflecting the heterogeneous distribution of plankton and seasonal productivity patterns, respectively. The attainment of neutral buoyancy helps these animals conserve energy and also reduces the chance of being detected by raptorial predators 12.

Temperature and pressure both affect the functioning of the lipid bilayers that constitute biological membranes 13. Marine organisms adapt to these physical constraints by changing the fatty acid composition of their cellular membranes 14. Lipids are also central to how metazoans survive food shortages 15 and attain neutral buoyancy 16. Recent work has specifically highlighted central roles for the polyunsaturated fatty acids (PUFAs) eicosapentaenoic acid (EPA, 20:5(n-3)) and docosahexaenoic acid (DHA, 22:6(n-3)) in buoyancy control, membrane functionality, and the starvation response of marine copepods 12,17–19. Pelagic zooplankton that feed in the euphotic zone principally derive EPA and DHA from ingested diatoms and flagellated microplankton, respectively. They are incapable of synthesising them de novo or by the elongation of dietary precursors at ecologically significant rates 20. Mesopelagic copepods, e.g. *Oithona* spp. and *Oncaea* spp., contain significant quantities of these PUFAs within their body tissues 21, and they likely play similar physiological roles. The origins of EPA and DHA in these animals remain unknown, in part reflecting the complexities associated with discerning the diets of mesopelagic copepods 22.

The majority of sinking organic matter supplied to heterotrophic organisms in the twilight zone is in the form of marine snow, faecal pellets, and other particles of detritus. These particles may have been previously ingested and reworked multiple times by zooplankton that selectively absorb the most labile and nutritious dietary compounds, including essential amino acids and PUFAs 17,23. This explains why concentrations of these compounds in suspended particles decline dramatically with depth 24–26, whereas the relative proportions of refractory polysaccharides and the C:N ratio of detritus both increase 27,28. The majority of organic matter available to twilight zone residents therefore consists of refractory organic particles that are devoid of compounds thought to be central to life in these waters. An exception to this occurs during the rapid sedimentation of phytoplankton cells, e.g. following diatom blooms, which are known to transport significant quantities of PUFAs down through the mesopelagic and into the abyss 25. However, the significance of these ephemeral events to twilight zone residents remains unknown. We suggest that deriving the majority of their annual metabolic- and nutritional demands from detritus poses potential problems for metazoans, which typically lack the endogenous enzymatic capacity for the degradation of long chain carbohydrates and biosynthesis of compounds such as EPA and DHA. Thus, zooplankton should in principle exploit any opportunity to enhance the acquisition of these compounds from their diet 29.

Animals that ingest a diet rich in refractory biopolymers, e.g. detritus or cellulose-rich leaves, typically display commensal or symbiotic relationships with intestinal microorganisms, perhaps best exemplified by the ruminant mammals 30. Some terrestrial and marine invertebrates also display digestive associations with bacteria 31,32. These animals have capacious, and often specialised guts that are capable of retaining food for prolonged periods. Pelagic copepods are known to harbour internal microorganisms 33,34, but the small size of those inhabiting the twilight zone suggests that bacterially-mediated digestion in their guts is unlikely 31,35.

## Microbial gardening

Zooplankton faecal pellets can contribute significantly to carbon export beneath the euphotic zone 3. Flux-feeding 36 copepods fragment, rather than ingest, the majority of encountered faecal material 37–41, and potentially explain why the number of faecal pellets observed beneath the euphotic zone is less than would be expected from the abundance of copepods in the overlying waters. The extension of this mechanism to include the fragmentation of all large detrital particles has recently helped reconceptualise the twilight zone carbon budget 4. However, the reasons underlying this seemingly wasteful strategy are obscure. Here we suggest that it offers significant energetic and nutritional benefit to the metazoans, despite losses through microbial respiration, which reduces the absolute quantity of available organic matter.

Fragmentation of large, detrital particles reduces their sinking speed 38, locally retaining organic resources that would otherwise sink into the abyss. It also increases their surface area, making them more amenable to bacterial colonisation and hence stimulates microbial pathways 10,38. The latter provides two distinct potential benefits to detritivores. Firstly, it exploits the extensive repertoire of bacterial exoenzymes for breaking down the plethora of refractory compounds supplied to the twilight zone, hence obviating the need for these enzymes in the detritivores. Indeed, this process may be a necessity because the degradation of long chain polysaccharides, e.g. cellulose, is enzymatically complex and almost exclusively undertaken by microorganisms 42. A second, major benefit of stimulating the microbial food web is the ‘trophic upgrading’ of the particulate organic matter: bacteria and heterotrophic protists are both capable of de novo synthesis of a range of labile and essential compounds, including EPA and DHA 29,34,43,44. The net result of fragmenting large, refractory particles of sinking organic matter is the production of slow-sinking particles that are hotspots of microbial activity. The resident flagellates and ciliates represent digestible and nutritious biomass that is of appropriate size for copepods to effectively harvest from the surface of particles.

Our assertion is supported by reports of smaller, slow-settling particles containing more labile compounds than larger ones 45,46. It is also consistent with the idea that twilight zone copepods only feed on a fraction of the total detrital pool 22, potentially selecting particle-attached bacteria 37 and microbes 40. The drawback to microbial gardening is that it ultimately reduces the absolute quantity of carbon available to the gardeners: attached microbes respire much of their substrate to CO_2_ and may be ingested by organisms that do not contribute to fragmentation.

The trade-off between maximising the direct ingestion of low-quality particulate organic detritus versus microbial gardening, i.e. the fragmentation of this material for colonisation by nutritionally-rich microbes that are subsequently ingested, was examined using an adapted version of a simple steady state model 4. The model follows the utilisation of sinking particles by bacteria and zooplankton and the subsequent cycling of carbon in the detrital food web along three pathways: colonisation and solubilisation of detritus by attached bacteria, consumption of detritus and resident microbial populations by detritivorous zooplankton, and the use of solubilisation products (dissolved organic carbon) by free-living bacteria. Detritus is divided into two types 47; large, fast-sinking particles (D1) and smaller, slower- or non-sinking particles (D2). The previous implementation of this model 4 assumed that detritivores have grazing access only to the former pool. During grazing, 30% (parameter *λ* = 0.3) was released as small, non-sinking material, a further 5% (parameter *r* = 0.05) being excreted as DOC (providing a growth substrate for free-living bacteria). The remainder was used with absorption (digestion) efficiency of 60% (parameter *β* = 0.6) and net production efficiency (fraction of absorption used for growth) of 39% (parameter *k* = 0.39) to give a combined growth efficiency of 0.23 (the product of *β* and *k*).

Without being able to graze on D2, the potential for microbial gardening was not investigated. We have modified the model by permitting detritivores to graze on the D2 detrital pool and its associated microbial biomass, and by altering key parameters to reflect the contrasting nutritional status of D1 and D2 for zooplankton (see Supporting Information Figs. S1 and S2 and Table S1). Whereas previously attached bacteria and zooplankton processed D1 equally (parameter *ψ*_B_ = 0.5) with 100% utilisation of D2 by bacteria, we now use *ψ*_B1_ and *ψ*_B2_ of 0.05 and 0.5 for D1 and D2, respectively, reflecting the active growth of microbial populations on D2 and greater zooplankton usage thereof. The efficiencies with which zooplankton utilise compounds and elements is variable 17,23 but we are unaware of any data relating specifically to twilight zone organisms. Model parameters are therefore chosen to illustrate our hypothesis and are not intended as definitive values.

Detritivorous zooplankton use large particles (D1) with a low absorption efficiency (*β*_1_ = 0.1) in accordance with the hypothesis that they do not have the capacity to digest the majority of this refractory material. The net production efficiency of absorbed D1 is also assigned a low value (*k*_1_ = 0.25) because of increased biosynthesis costs resulting from the nutritional imbalance of compounds in food relative to the requirements of the detritivores 48. Organic matter in D2, mostly represented by labile and nutritious microbial biomass, is easier to absorb (*β*_2_ = 0.5) and more closely matches the nutritional requirements of the detritivores. The lower costs of biosynthesis give rise to increased net production efficiency (*k*_2_ = 0.5).

**Figure 2:**
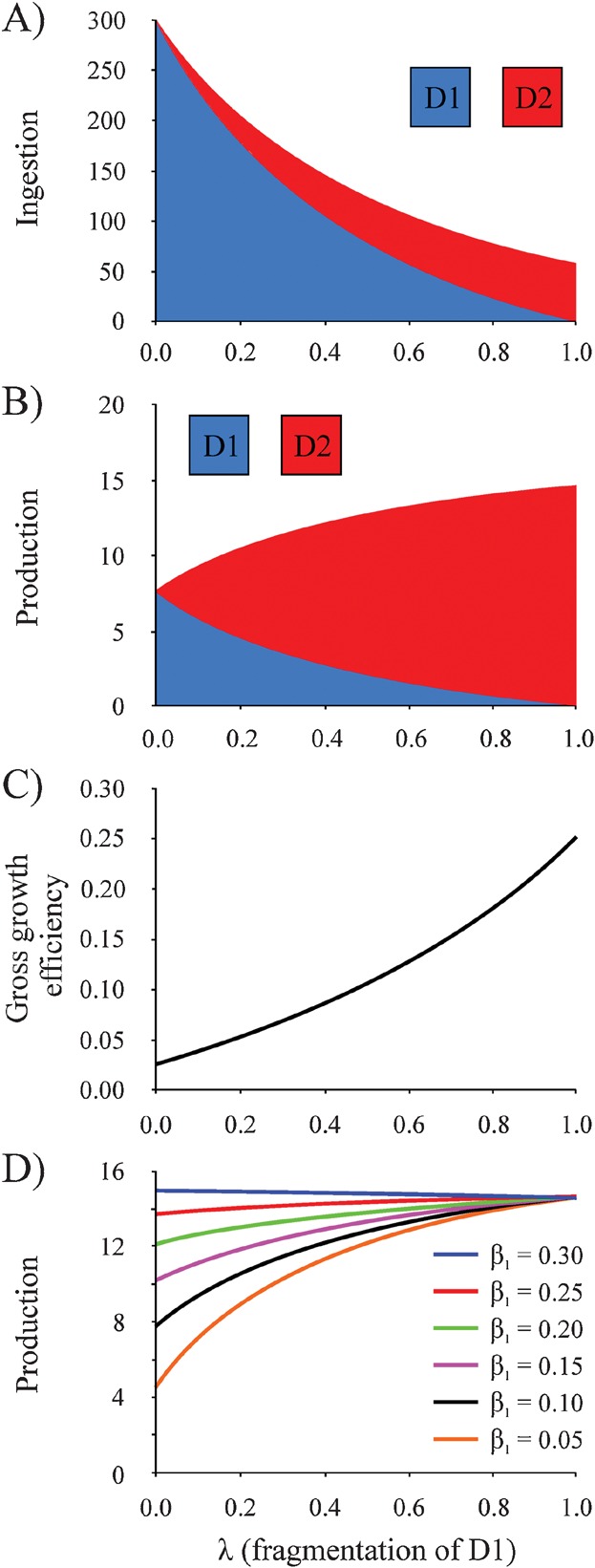
Model-predictions illustrating how the proportion of large, fast sinking particles (D1) fragmented into smaller, slow- or non-sinking particles (D2) by detritivorous zooplankton (*λ*) affects their: A: Ingestion, B: Production, C: Gross growth efficiency, and D: Production when the absorption efficiency of D1 particles (*β*_1_) is varied. The complete budget is presented in Supporting Information Fig. S3. Ingestion and production (panels A, B, and D) are scaled to relative to a detrital flux of 100 (nominal units) entering the twilight zone.

The model is clearly a simplification of the full complexity of the twilight zone and represents complex and likely variable terms, e.g. detritus lability, bacterial and zooplankton physiology and ecology, as crude averages in this vertically expansive ecosystem. Nevertheless, it enables us to examine the potential of microbial gardening as a strategy for maximising the growth of detritivorous zooplankton by varying the fraction of D1 fragmented to D2 during grazing (parameter *λ*). As *λ* increases, a greater proportion of D1 detritus is fragmented, favouring the ingestion of D2 detritus by zooplankton, the proposed gardening pathway. Ingestion decreases overall (Fig. 2A) because, quantitatively, much of the D2 detrital pool is lost to CO_2_ via microbial respiration. Production is lowest when detritivores ingest, rather than fragment, D1 (*λ* = 0; Fig. 2B), even though ingestion is greatest. This reflects the low efficiencies with which they can absorb and subsequently utilise this nutritionally inferior material for the production of biomass. Fragmentation of D1 (*λ* > 0) increases detritivore production because the resulting crop of microbial biomass growing on D2 consists of compounds that are more easily absorbed and assimilated into biomass. Increasing *λ*, and hence microbial gardening, therefore serves to increase the gross growth efficiency of the detritivores (Fig. 2C). The benefits of gardening decline as D1 detritus becomes easier to absorb, i.e. its lability is increased (Fig. 2D). It ultimately becomes an adverse strategy when *β*_1_ ≥ 0.3. The utility of gardening will therefore vary in space, both vertically and horizontally, and also in time, depending on the numerous processes that influence the biochemical makeup of detritus.

## Conclusions and prospects

Microbial gardening offers a potentially successful trophic strategy for zooplankton in the twilight zone of the ocean. It provides a source of easily digestible biomass that is rich in essential micronutrients. The model captures these benefits by assuming carbon gross growth efficiency increases when feeding on microbe-infested detritus. A more detailed analysis of this concept could in principle be carried out using a stoichiometric model with multiple currencies representing carbon and other nutritional factors. Taking into account both growth requirements and basal metabolic demands associated with tissue turnover, the limiting substrate can be identified, and growth predicted accordingly 49. The conceptualisation of such models requires an understanding of the subject matter's physiology and empirical data for their parameterisation. In reality, we know little about the roles of micronutrients in deep-dwelling zooplankton or how they attain them.

We have much to learn about the biochemistry of different particle classes in the twilight zone, the physiological adaptations that facilitate metazoan life in this environment and the ecological interactions that provision the resident organisms with the necessary biochemical compounds. Our ability to quantitatively understand the significance of microbial gardening in the twilight zone, and the wider roles of the resident detritivorous zooplankton, requires more detailed knowledge of the food consumed by these animals, their basal metabolic demands, and the efficiencies with which they absorb and assimilate elements and nutrients from their daily ration. These are not trivial challenges, given the small size of subject organisms and the potential for artefacts associated with capturing and removing them from their natural environment for experimentation. It is probable that a combination of in-situ preservation techniques, proxy-based estimates of physiology, and molecular tools, e.g. transcriptomics, proteomics, and metabolomics, will provide further insight into the role of zooplankton in the twilight zone.

## Abbreviations

BCP: biological carbon pump
DHA: docosahexaenoic acid (22:6(n-3))
EPA: eicosapentaenoic acid (20:5(n-3))
PUFA: polyunsaturated fatty acid
