# Supplementary material for: Microbial gardening in the ocean's twilight zone: Detritivorous metazoans benefit from fragmenting, rather than ingesting, sinking detritus
Source: Bioessays. 2014 Sep 12;36(12):1132–7. doi: 10.1002/bies.201400100 (PMC4278546; doi:10.1002/bies.201400100)
Supplement: Supplementary file 1 [file bies0036-1132-SD1.docx]

**Microbial gardening in the ocean’s twilight zone: Detritivorous metazoans benefit from fragmenting, rather than ingesting, sinking detritus**

Daniel J. Mayor*, Richard Sanders, Sarah L.C. Giering, Thomas R. Anderson

* [dan.mayor@abdn.ac.uk](mailto:dan.mayor@abdn.ac.uk)

**Model description**

The model is a steady state flow analysis of C cycling within the foodweb of the twilight zone (Fig. S1). We assume this environment begins at 50m beneath the surface and extends vertically downwards to 1000m (Giering et al., 2014). Carbon flows through multiple nodes within the foodweb, with parameters specifying the fraction of material being channelled along the different pathways at each node. Stocks are not modelled. The original concept was developed by Anderson and Tang (2010) and modified by Giering et al. (2014; hereafter G14). We provide a brief description of the model here, highlighting further modifications specific to our analysis of microbial gardening as a growth strategy for detritivorous zooplankton. The model is coded in R and available on request to T.R. Anderson ([tra@noc.ac.uk](mailto:tra@noc.ac.uk)).

In the model, particulate organic carbon (POC) enters the twilight zone as sinking detritus (for ease of analysis, an assumed flux of 100 is used; the units are irrelevant although a characteristic flux is 68 g C m^-2^ d^-1^: Giering et al., 2014). The biological processing and eventual release of this detrital organic carbon as CO_2_ is traced along three pathways: colonisation and solubilisation of detritus by attached bacteria, consumption of detritus and resident microbial populations by detritivorous zooplankton, and the use of solubilisation products (dissolved organic carbon) by free-living bacteria. A brief description follows, with parameter values listed in Table S1. Note that this is a hypothetical exercise. The parameter values are consistent with our current, albeit poor, understanding of the twilight zone, as outlined in the main text. We demonstrate that, using these values, microbial gardening is a quantitatively superior growth strategy for detritivorous zooplankton.

The key to understanding our application of the model is in how it handles the processing of detritus by bacteria and zooplankton. Detritus is divided into two types, fast-sinking (large) particles, D1, and slow-sinking (small) particles, D2. For the purpose of this (hypothetical) exercise, we assume that all sinking POC that enters the twilight zone is D1 and show that if this carbon is broken up and released, rather than ingested, this favours zooplankton production. In reality, as much as 60% of POC entering the twilight zone may be as slow-sinking detritus (Alonso‐González et al., 2010; Riley et al., 2012). Excluding the input of D2 into the twilight zone in our analysis has no bearing on our conclusions with respect to microbial gardening.

The fractional consumption of D1 and D2 by microbes is specified by parameters ψ_B1_ and ψ_B2_. In G14, ψ_B1_ was set to 0.5 (i.e. equal consumption by attached bacteria and detritivores) and consumption of D2 was solely by bacteria (in effect, ψ_B2_ = 1.0). Here, we used ψ_B1_ = 0.05 and ψ_B2_ = 0.50 (permitting access to D2 by detritivores). The low value of ψ_B1_ = 0.05 is consistent with the idea that the majority of D1 carbon cannot be rapidly utilised by microbes because it is refractory and less accessible owing to the low surface area:volume ratio of these large particles. Carbon in the D2 pool, which has a greater surface area:volume ratio, is more favourable in this respect. In G14, fraction 1-ψ_B1_ was consumed by zooplankton but here we introduce a new parameter, ψ_Z_, for this purpose, assigning a value of 0.8. Fraction 1-ψ_B1_-ψ_Z_ is now lost, assumed exported out of the base of the twilight zone. There is no such loss for D2 and utilisation by zooplankton is 1-ψ_B2_.

Of the detrital carbon processed by attached bacteria, 50% is solubilised to dissolved organic carbon (DOC) (parameter α) with the remainder used for growth with efficiency 0.24 (parameter ω_att_; the balance is respired to CO_2_). Attached bacteria are grazed by bacterivores with release via DOC, faecal pellets (to D2) and respiration controlled by parameters r_V_, β_V_ and k_V_ (in the same way as for detritivores: see below). DOC is processed by free-living bacteria with growth efficiency ω_fl_, with associated consumption by bacterivores, with parameters the same as those of attached microbes (r_V_, β_V_ and k_V_).

The processing of detritus D1 by zooplankton is illustrated in Fig. S2. Fraction (1-ψ_B1_) of detritus loss is due to grazing, and associated with it (1-ψ_B1_)ζ of associated microbial populations (fraction 1-ζ goes to bacterivores). Of the encountered D1, fraction λ is immediately broken up and released as D2 particles, encouraging microbial gardening. Parameter λ was varied between 0 and 1. The material not broken up is passed into the gut and subject to absorption efficiency β_1_. Fraction 1-β_1_ is voided as faecal pellets (to D1). Growth is then fraction k_1_ of absorbed C, with the remainder respired as CO_2_. D2 (and associated microbes) is handled by detritivores in the same way as D1 except that there is no breakup of particles at the outset. Associated parameters for absorption and net production efficiencies are β_2_ and k_2_ respectively. Parameter values were adjusted from those of G14 in order to be consistent with the notion that D1 detritus represents an inferior metabolic substrate compared to D2. Absorption efficiencies were set at 0.05 and 0.50 for β_1_ and β_2_ respectively, reflecting the difficulty associated with digesting the former. Likewise, net production efficiencies of 0.25 and 0.50 were set for D1 and D2 respectively, a result of the greater nutritional imbalance likely to be encountered in substrates absorbed from D1. Finally, the model is closed with an infinite loop of carnivorous zooplankton that consume both detritivores and free-living bacterivores. Their growth and associated loss terms are specified using parameters r_Z_, λ_Z_, β_Z_ and k_Z_.

The results presented in the main text illustrate how ingestion, production and associated GGE increases as the fraction of D2 detritus increases in the diet of detritivorous zooplankton (Fig. 2). As a further supplementary analysis, we compare in more detail the predicted C budget for detritivores for λ=0 (no microbial gardening) and λ=1 (maximum microbial gardening) (Fig. S3). When λ=0, zooplankton ingest D1 and, due to its refractory nature, void most of it as faecal pellets, also D1, creating a repeating loop. It appears to be a wasteful and ineffective growth strategy, reflected as low gross growth efficiency. In contrast, when D1 is broken up and released as D2 (λ=1), production is higher despite the overall lower intake. The intake of microbes is also higher.

**Figures**

**Figure S1.** Flow diagram illustrating carbon cycling pathways in the model. Flows to small coloured circles enter sinking detritus (D1; brown), suspended detritus (D2, cyan), DOC (pink) and CO_2_ (yellow). Attached and free-living microbes in the model are separated into two trophic levels, bacteria and bacterivores.

**Figure S2.** Processing of detritus D1 and associated microbes by zooplankton.

**Figure S3.** Model-predicted flows of carbon illustrating the differences between no fragmentation (λ=0) and complete fragmentation (λ=1) of D1 detritus by metazoan detritivores. The model is initiated assuming that 100 units of D1 enter the twilight zone.

**Supplementary Table S1**. Model parameters and default values (note, all parameters are dimensionless).

| **Parameter** | **Description** | **Value** |
| --- | --- | --- |
| ψ_B1_ | partitioning of D1 to attached bacteria | 0.05 |
| ψ_B2_ | partitioning of D2 to attached bacteria | 0.50 |
| ψ_Z_ | Partitioning of D1 to zooplankton | 0.80 |
| α | solubilisation losses: attached bacteria | 0.5 |
| ω_att_ | BGE: attached bacteria | 0.24 |
| ω_fl_ | BGE: free-living bacteria | 0.08 |
| r_V_ | release of DOC as excretion by bacterivores | 0.05 |
| r_Z_ | release of DOC as excretion by carnivores | 0.05 |
| λ | grazing losses D1 to D2: detritivores | 0 - 1 |
| λ_Z_ | grazing losses to D2: carnivores | 0.15 |
| β_V_ | absorption efficiency: bacterivores | 0.72 |
| β_1_ | absorption efficiency: detritivores on D1 | 0.10 |
| β_2_ | absorption efficiency: detritivores on D2 | 0.50 |
| β_Z_ | absorption efficiency: carnivores | 0.66 |
| k_V_ | NPE: bacterivores | 0.44 |
| K_1_ | NPE: detritivores on D1 | 0.25 |
| K_2_ | NPE: detritivores on D2 | 0.50 |
| k_Z_ | NPE: higher zooplankton | 0.39 |
| ζ | particle microbial losses to detritivores | 0.48 |

**References**

**Alonso‐González IJ, Arístegui J, Cindy Lee C, Sanchez‐Vidal A,** et al., 2010. Role of slowly settling particles in the ocean carbon cycle. *Geophys Res Lett* **37**: L13608, doi:10.1029/2010GL043827.

**Anderson TR, Tang KW.** 2010. Carbon cycling and POC turnover in the mesopelagic zone of the ocean: Insights from a simple model. *Deep Sea Res II* **57**: 1581-1592.

**Giering SLC, Sanders R, Lampitt RS, Anderson TR,** et al. 2014. Reconciliation of the carbon budget in the ocean’s twilight zone. *Nature* **507**: 480-483.

**Riley JS, Sanders R, Marsay C, Le Moigne FAC,** et al. 2012). The relative contribution of fast and slow sinking particles to ocean carbon export. *Global Biogeochem Cycles* **26**: GB1026, doi:10.1029/2011GB004085.
